# Electrochemically Synthesized Poly(3-hexylthiophene) Nanowires as Photosensitive Neuronal Interfaces

**DOI:** 10.3390/ma14164761

**Published:** 2021-08-23

**Authors:** Szilveszter Gáspár, Tiziana Ravasenga, Raluca-Elena Munteanu, Sorin David, Fabio Benfenati, Elisabetta Colombo

**Affiliations:** 1 Electrochemistry Laboratory, International Centre of Biodynamics, 060101 Bucharest, Romania; rmunteanu@biodyn.ro (R.-E.M.); sdavid@biodyn.ro (S.D.); 2 Center for Synaptic Neuroscience and Technology, Istituto Italiano di Tecnologia, 16132 Genova, Italy; Tiziana.Ravasenga@iit.it (T.R.); Fabio.Benfenati@iit.it (F.B.); 3 IRCCS Ospedale Policlinico San Martino, 16132 Genova, Italy

**Keywords:** poly(3-hexylthiophene), nanowires, template-assisted electrochemical synthesis, biocompatibility, neuronal interface

## Abstract

Poly(3-hexylthiophene) (P3HT) is a hole-conducting polymer that has been intensively used to develop organic optoelectronic devices (e.g., organic solar cells). Recently, P3HT films and nanoparticles have also been used to restore the photosensitivity of retinal neurons. The template-assisted electrochemical synthesis of polymer nanowires advantageously combines polymerization and polymer nanostructuring into one, relatively simple, procedure. However, obtaining P3HT nanowires through this procedure was rarely investigated. Therefore, this study aimed to investigate the template-assisted electrochemical synthesis of P3HT nanowires doped with tetrabutylammonium hexafluorophosphate (TBAHFP) and their biocompatibility with primary neurons. We show that template-assisted electrochemical synthesis can relatively easily turn 3-hexylthiophene (3HT) into longer (e.g., 17 ± 3 µm) or shorter (e.g., 1.5 ± 0.4 µm) P3HT nanowires with an average diameter of 196 ± 55 nm (determined by the used template). The nanowires produce measurable photocurrents following illumination. Finally, we show that primary cortical neurons can be grown onto P3HT nanowires drop-casted on a glass substrate without relevant changes in their viability and electrophysiological properties, indicating that P3HT nanowires obtained by template-assisted electrochemical synthesis represent a promising neuronal interface for photostimulation.

## 1. Introduction

P3HT is surrounded by great interest mainly because of its excellent optoelectronic properties (such as good electrical conductivity and high extinction coefficient) and good processability, which make this polymer an excellent choice for building organic optoelectronic devices (e.g., organic solar cells). As a consequence of the interest surrounding P3HT, several ways to synthesize this polymer were developed and described in the literature. Most of these ways are based on chemical synthesis [1]. However, few electrochemical syntheses of P3HT films have also been described [2,3,4,5,6]. A study comparing chemical and electrochemical approaches to obtain P3HT highlights that electrochemical synthesis gives a P3HT characterized by better electrical conductivity and a higher degree of crystallinity [2].

It is now widely accepted that nanostructuring provides materials, including optoelectronic materials [7], with improved or completely new properties, which, in turn, might facilitate new applications. Polymer solar cells based on P3HT and [6,6]-phenyl-C61-butyric acid methyl ester (PCBM) were found to be more efficient when made of a dense network of crystalline P3HT nanowires than when made of amorphous P3HT [8,9]. The photoluminescence of individual semicrystalline P3HT nanoparticles also supports the hypothesis that hierarchical assemblies of conducting polymer nanoparticles could be a way to achieve higher efficiency polymer solar cells [10]. As yet another example, P3HT nanowires doped with 2,3,5,6-tetrafluoro-7,7,8,8-tetracyanoquinodimethane were found to be characterized by electrical resistivities 2–4 orders of magnitude smaller than the corresponding P3HT films [11]. As a consequence, several ways of obtaining P3HT nanostructures were also developed. P3HT nanoparticles were obtained by a reprecipitation method, exploiting the different solubility of P3HT in, for example, ethanol and chloroform [12]. Nanoscale P3HT aggregates were also obtained by dissolving P3HT at higher temperatures (e.g., 70 °C) and subsequently cooling the solution (e.g., to 38 °C, with 1 °C min^−1^) [13,14]. As yet another possibility, solid P3HT was “melted” into the cylindrical pores of a nanoporous alumina membrane in order to obtain P3HT nanowires [11,15,16]. P3HT nanowires were also obtained by drying a solution of P3HT into the pores of a nanoporous alumina membrane [17]. A top-down, photopatterning-based method to obtain P3HT nanowires (20–70 nm thickness, 200–900 nm width, and 40 µm length) was also reported [18]. Finally, template-assisted, electrochemical synthesis of P3HT nanostructures was also described a couple of times [3,5]. The cylindrical pores of nanoporous alumina membranes are used as a template by this procedure as well. However, unlike all other procedures to obtain P3HT nanostructures, this procedure uses a 3HT monomer solution instead of using a preexisting polymer solution. The monomer is loaded into the cylindrical pores of the template and then turned into polymer by electrochemical oxidation (i.e., by oxidative electropolymerization). The procedure can be tuned to give either nanowires or nanotubes [5]. Compared to the other procedures to make P3HT nanostructures, the template-assisted electrochemical synthesis has several advantages. First, it starts with 3HT (instead of P3HT) and turns 3HT into P3HT while completely avoiding both long reaction times and toxic transition metal catalysts [6]. Second, it provides excellent control over the length and diameter of the synthetized nanowires (via the dimensions of the template and via the concentration of monomer and the amount of charge used during the synthesis) [5]. Third, the procedure allows for building multisegmented nanowires in which polymer segments are combined with segments made of metal or metal oxide (as demonstrated with poly(pyrrole) [19]). Multisegmented nanowires can perform multiple functions simultaneously. None of the other procedures to make P3HT nanostructures have such a feature.

In a more recent development, P3HT was also integrated into different neuronal interfaces, with the main aim being to facilitate photostimulation of neuronal activity. For example, planar P3HT films and P3HT nanoparticles were shown to facilitate the restoration of the light sensitivity in retinal neurons and, thus, the recovery of visual functions in a rodent model of retinal degeneration [20,21]. A photosensitive neural interface that combines P3HT and an organohalide perovskite was also reported [22]. The photosensitivity of this interface was attributed to the organohalide perovskite, however, P3HT had the important role of improving the stability and the biocompatibility of the device (as the perovskite was both unstable and toxic). Another study compared neural interfaces in which P3HT was deposited onto graphene with neural interfaces in which P3HT was deposited onto a film of poly(3,4-ethylenedioxythiophene) and poly(styrenesulfonate) (PEDOT:PSS) [23]. A stronger light-transduction efficiency was observed for the graphene-based interfaces. The use of conjugated polymers (including P3HT) for implantable electronics was very recently reviewed elsewhere [24].

Motivated by the above highlighted biomedical applications of P3HT, by the paucity of data on the template-assisted, electrochemical synthesis of P3HT nanowires, and by the above-detailed advantages of this method of making nanowires, the present study had two important objectives. First, it aimed to investigate the template-assisted, electrochemical synthesis of P3HT nanowires. Second, it also aimed to assess the biocompatibility of such nanowires and to study the effect of such nanowires on the physiological properties of neurons. The obtained results show that photosensitive P3HT nanowires of different and controlled lengths can be relatively easily synthesized using template-assisted, electrochemical synthesis. They also show that the electrochemically synthesized P3HT nanowires are biocompatible and do not affect the electrophysiological properties of neurons. Taken together, the results prove the potential of this nanomaterial as a component of advanced neural interfaces which allow for the photostimulation of neurons.

## 2. Materials and Methods

### 2.1. Materials

3HT, TBAHFP, propidium iodide solution (1 mg ml^−1^), DNase, poly(L-lysine), phosphate buffered saline (PBS) tablets, bovine serum albumin (BSA), Triton X-100, Hoechst-33342, Mowiol and common chemicals (e.g., NaCl, CaCl_2_, etc.) were purchased from Merck/Sigma-Aldrich (Darmstadt, Germany). CuCl_2_·2H_2_O was purchased from Carl Roth GmbH (Karlsruhe, Germany). NaOH was purchased from Lach-Ner S.R.O. (Neratovice, Czech Republic). Acetonitrile was purchased from Scharlau S.L. (Barcelona, Spain). Metallic Cu (99.98%) was purchased from Kurt J. Lesker Company (Pittsburgh, PA, USA). Orotemp 24 gold plating solution was purchased from Italgalvano S.P.A. (Lodi Vecchio, Italy). CuSO_4_·5H_2_O, KI, metallic I_2_, and 37% HCl solution were purchased from smaller Romanian providers. Porous alumina membranes were purchased from Whatman International Ltd. (Maidstone, UK). D-Tube Dialyzer Maxi (MWCO 3.5 kDa) was from Merck (Darmstadt, Germany). Borosilicate glass capillaries were purchased from Kimble (Queretaro, Mexico). Neurobasal medium, GlutaMax, penicillin–streptomycin, B27, secondary antibody Alexa488, anti β3 Tubulin and glass coverslips were purchased from Thermo Fischer Scientific (Waltham, MA, USA). Cyanquixaline (CNQX), bicuculline methiodide and CGP58845 were purchased from Tocris (Bristol, UK). C57BL6J mice were purchased from Charles River (Calco, Italy). All chemicals were used as purchased. All aqueous solutions were made using ultrapure water from a Direct-Q 3 UV water purification system from Millipore S.A.S. (Molsheim, France).

### 2.2. Template-Assisted Electrochemical Synthesis of P3HT Nanowiresaterials

**Figure 1:**
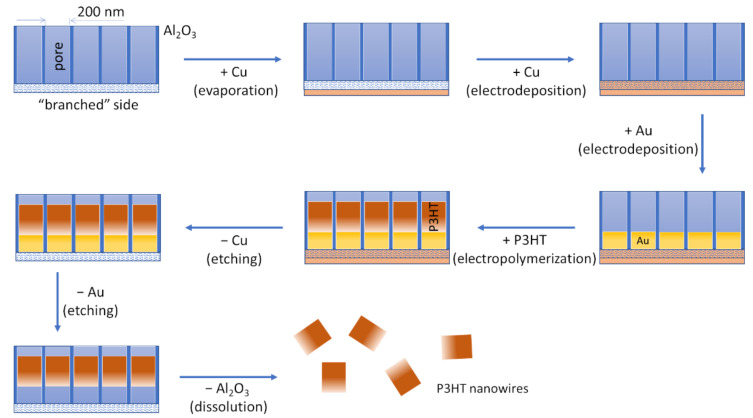
Schematic representation of the template-assisted, electrochemical synthesis of P3HT nanowires. A nanoporous alumina membrane is used as template. After covering the “branched” side of the membrane with Cu by physical vapor deposition, three electrochemical steps are carried out, namely, electrodeposition of Cu, electrodeposition of Au, and electropolymerization of P3HT. Following these three electrochemical steps, the template is disassembled by using two metal etching solutions (one to remove Cu and one to remove Au) and a NaOH solution (to dissolve the alumina). A suspension of P3HT nanowires is obtained. The nanowires are then washed until a neutral pH suspension is obtained (not shown).

The procedure to obtain the P3HT nanowires is based on a procedure previously used by our group to produce poly(pyrrole)-metal nanorods [25,26,27] and is schematically depicted in Figure 1. Porous alumina membranes (featuring “branched” pores continued by cylindrical pores with diameters around 200 nm) were employed as the template for nanowire growth. A 400 nm thick layer of Cu was deposited onto the side with the “branched” pores of such membranes by physical vapor deposition carried out using a PVD 75 system (from Kurt J. Lesker Company, Pittsburgh, PA, USA). This layer of Cu served as the working electrode in the subsequent three electrochemical steps carried out using a PGSTAT128N potentiostat (from Metrohm Autolab B.V., Utrecht, The Netherlands). A Pt wire was used as the counter electrode, and an Ag wire coated with AgCl was used as the reference electrode. In the first electrochemical step, Cu was plated into the “branched” pores of the membrane by using a solution of 1 M CuSO_4_ in water, an applied potential of −0.5 V, and ~21 C of charge. In the second electrochemical step, short Au segments were grown into the cylindrical pores of the membrane by using Orotemp 24 plating solution, an applied potential of −0.9 V, and ~2 C of charge. In the last electrochemical step, P3HT nanowires were electropolymerized into the cylindrical pores of the membrane by using a solution of 11 mM 3HT and 100 mM TBAHFP in acetonitrile, two potential cycles in between −0.25 V and +2 V, and a potential scan rate of 0.05 V s^−1^. These experimental conditions allowed for obtaining “short” P3HT nanowires. “Long” P3HT nanowires were obtained by increasing the 3HT concentration from 11 mM to 30 mM while maintaining all the other experimental conditions unchanged. After these three electrochemical steps, the membrane was immersed first into a solution of 0.5 M CuCl_2_ in 20% HCl (to etch away the Cu layers), then into a solution of 0.6 M KI and 0.1 M I_2_ in water (to etch away the Au segments), and, finally, into a solution of 1 M NaOH in water (to dissolve the alumina membrane and release the embedded P3HT nanowires). Subsequently, the P3HT nanowires were washed several times with ultrapure water (using centrifugation to separate the nanowires from the solution after each washing step). Finally, the nanowires were suspended in ultrapure water. The mass concentration of the resulting P3HT nanowire solutions was in the range of hundreds of µg mL^−1^ (but currently still characterized by significant dispersion due to the high number of washing steps which followed the synthesis). “Long” P3HT nanowires were observed to aggregate less when they were suspended in ethanol than when they were suspended in water. Conclusions presented in this work are based on 3 batches of “long” P3HT nanowires and 5 batches of “short” P3HT nanowires.

### 2.3. Characterization of the P3HT Nanowires

The P3HT nanowires were characterized by atomic force microscopy (AFM), scanning electron microscopy (SEM), energy-dispersive X-ray spectroscopy (EDS), Raman spectroscopy, UV-vis spectroscopy and by measuring the photocurrents produced by the illumination of glassy carbon electrodes modified with the P3HT nanowires.

The AFM study was carried out using a Nanowizard II instrument from JPK Instruments A.G. (Berlin, Germany) and with the P3HT nanowires drop-casted onto glass coverslips. The AFM images were obtained in air and in intermittent contact-mode using line rates as slow as 0.2 Hz and NCSTR AFM probes (from Nano World A.G. (Neuchâtel, Switzerland), with cantilevers characterized by a resonance frequency around 160 kHz and a force constant of 7.2 N m^−1^). The ratio between the set-point amplitude and the free amplitude of the AFM cantilever was set to 0.5–0.6. The obtained AFM images were used to determine both the lengths and the diameters of our nanowires. Important to keep in mind, the dimensions of the nanowires are somewhat distorted due to the combination of the tip and sample geometries (i.e., by convolution, as described, for example, in [28]).

To characterize the nanowires by SEM and Raman spectroscopy, they were drop-casted onto glass coverslips. The resulting samples were characterized first by an analytical (low-vacuum) SEM using a JEOL JSM-6490LA (JEOL, Akishima, Japan) operating at 20 kV acceleration voltage (5 kV for imaging). An amorphous carbon coating was sputtered onto the samples to avoid overcharging during acquisition. EDS (JEOL, Akishima, Japan; EX-230) was used to evaluate the presence of species which might have contaminated the nanowires during their synthesis and processing.

Raman spectra were acquired with a Raman microscope from Renishaw (Wotton-under-Edge, UK). The samples were excited at 633 nm to avoid the strong background generated by the autofluorescence of P3HT when excited in the green range of the spectrum. Excitation and Raman signal collection were performed with a 50× air objective (NA = 0.7). Acquisition time of the spectra was set at 1 s.

The UV-vis spectra of the P3HT nanowire suspensions were obtained using an Evolution 600 UV-vis spectrophotometer and the associated Vision Pro software (both from Thermo Fisher Scientific, Waltham, MA, USA). The raw spectra were corrected with the spectrum of the used solvent (i.e., with the spectrum of either water or ethanol) and normalized by dividing the absorbance value at each wavelength by the maximum absorbance value. This facilitated an easy comparison of the spectra of the different P3HT nanowire suspensions (even though, as already mentioned above, the high number of washing steps used to remove template residues translated into suspensions with somewhat different concentrations of P3HT nanowires).

In order to observe whether the electrochemically synthesized P3HT nanowires are characterized by photosensitivity or not, we investigated the photocurrents generated by a disk-shaped, glassy carbon electrode (2 mm in diameter, from Metrohm A.G., Herisau, Switzerland) modified with the P3HT nanowires. The electrode was modified with the nanowires by a simple drop-casting procedure in which aliquots of 3 µL of nanowire suspension were successively placed onto the electrode (as soon as the previous aliquot evaporated). Photocurrents were recorded three times: before the modification of the electrode with the nanowires, after the modification of the electrode with a total volume of 18 µL of nanowire suspension, and, finally, after the modification of the electrode with a total volume of 36 µL of nanowire suspension. To measure photocurrents, the nanowire-modified electrode (used as working electrode) was completed with a Pt counter electrode and an Ag/AgCl, 3M KCl reference electrode (both from Metrohm A.G., Herisau, Switzerland). The three-electrode system was then immersed into PBS solution and the potential of the nanowire-modified electrode set to 0 V *versus* the open circuit potential. The nanowire-modified electrode was then periodically illuminated with a white light LED placed at 2 cm from the electrode surface (i.e., with a power density of ~1.3 mW cm^−2^ as measured with a 3A-P-V1 sensor connected to a computer via a Juno USB interface, both from Ophir Photonics, Jerusalem, Israel). The photocurrents were recorded for 11 min with a temporal resolution of 0.5 s.

### 2.4. Primary Neuronal Cultures

All experiments were performed in accordance with the guidelines and regulations of the Italian Ministry of Health (authorization no. 306/2016-PR). Cultures were obtained from cerebral cortices derived from embryonic day 18 C57BL6J mice. Briefly, pregnant mice were sacrificed by CO_2_ inhalation, and 18-day embryos (E18) were removed by cesarean section. Cortices were dissociated by enzymatic digestion in 0.25% trypsin containing 0.25 mg mL^−1^ DNase for 30 min, at 37 °C, and then triturated with a fire-polished Pasteur pipette. No antimitotic drugs were added to prevent glia proliferation. Neurons were plated on poly(l-lysine) coated glass coverslips (ctrl). Two to six µL of P3HT nanowires suspension in absolute ethanol were drop casted onto poly(l-lysine) coated glass coverslips (NWs) and dried before neurons plating. Cultures were incubated at 37 °C, 5% CO_2_, 90% humidity in medium consisting of Neurobasal medium supplemented with 2 mM GlutaMax, 100 U mL^−1^ penicillin-streptomycin, and 2% B27.

### 2.5. Cell Viability

Cortical neurons were seeded onto poly(l-lysine)-modified glass coverslips (ctrl) and onto poly(l-lysine)- and P3HT nanowire-modified glass coverslips (NWs). Live cells were stained with propidium iodide for cell death quantification and Hoechst-33342 (1 µM) for nuclei visualization. Cell viability was quantified at 20× (0.5 NA) magnification using a Nikon Eclipse-80i upright epifluorescence microscope (Nikon, Tokyo, Japan), with random sampling of 3 to 8 fields per sample. Image analysis was performed using the ImageJ open-source software (version 1.53c) and the Cell Counter plugin.

### 2.6. Immunocytochemistry and Confocal Microscopy

Primary cortical neurons cultured on P3HT nanowire-modified glass coverslips were fixed in 4% paraformaldehyde in PBS solution for 12 min. After being washed in PBS solution, samples were permeabilized in 0.2% Triton X-100 for 5 min, then incubated with BSA (2%) for 30 min to prevent nonspecific binding. The immunostaining was performed by sequential incubation with anti β3 tubulin primary and Alexa 488 secondary antibodies in BSA 2%. For cell nuclei visualization, samples were incubated in Hoechst-33342 (1 mM) for 5 min. Coverslips, mounted in Mowiol fluorescent mounting medium, were observed using a Leica TCS SP8 confocal microscope (Leica, Wetzlar, Germany).

### 2.7. Patch-Clamp Recordings

Mouse cortical neurons were recorded at 12–15 days after plating. Patch pipettes, prepared from thin borosilicate glass capillaries, were pulled to a final resistance of 4–6 MΩ when filled with standard internal solution containing (in mM) 126 K gluconate, 4 NaCl, 1 MgSO_4_, 0.02 CaCl_2_, 0.1 BAPTA, 15 glucose, 5 HEPES, 3 ATP, and 0.1 GTP (pH 7.3 with KOH). All experiments were performed at room temperature (18–24 °C). Data acquisition was performed using PatchMaster program (HEKA Elektronik, Reutlingen, Germany). Current-clamp recordings were performed at a holding potential of −65 mV, and action potential firing was induced by injecting current steps of 10 pA lasting 1 s. Cells were maintained in extracellular standard solution (Tyrode) containing (in mM): 140 NaCl, 2 CaCl_2_, 1 MgCl_2_, 4 KCl, 10 glucose, and 10 HEPES (pH 7.3 with NaOH), in which D-AP5 (50 μM), CNQX (10 μM), bicuculline methiodide (30 μM), and CGP58845 (5 μM) were added to block NMDA, non-NMDA, GABAA, and GABAB receptors, respectively.

## 3. Results and Discussion

### 3.1. Template-Assisted Electrochemical Synthesis of P3HT Nanowires

Several recent reviews (e.g., [29,30]), each based on hundreds of scientific papers, indicate that the template-assisted electrochemical synthesis of nanowires is an effervescent field of research. Although this method already facilitated obtaining a myriad of metal, metal oxide, and polymer nanowires, it was seldom used to make P3HT nanowires. This fact, together with the great potential carried by P3HT nanowires in the development of organic optoelectronic devices and neural interfaces, motivated the present study.

**Figure 2:**
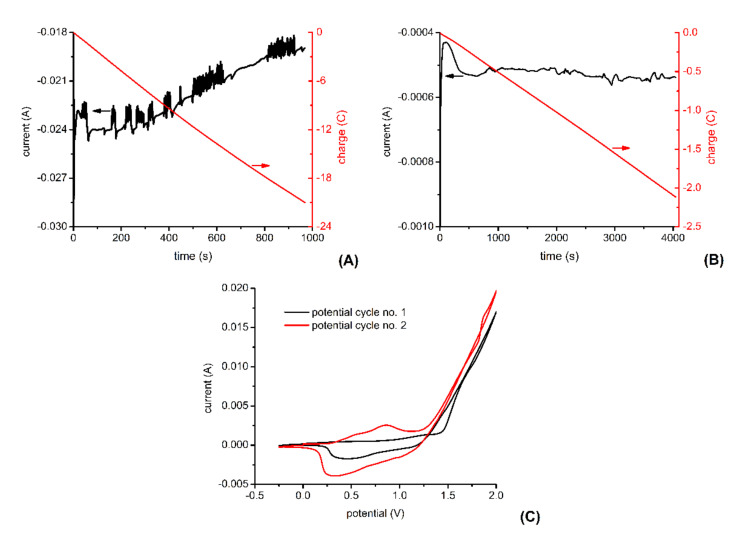
Electrochemical signals recorded during: deposition of Cu into the “branched” pores of the alumina template (**A**), deposition of Au segments into the cylindrical pores of the alumina template (**B**), and electropolymerization of P3HT into the cylindrical pores of the alumina template (**C**). Noise affecting current signals is due to occasionally “stirring” the solution during the electrochemical deposition. See the Materials and Methods section for additional experimental details.

Figure 2 shows typical electrochemical signals observed during the template-assisted electrochemical synthesis of P3HT nanowires. As shown in Figure 2A, the electrochemical deposition of metallic Cu into the “branched” pores of the alumina template produces a current which increases from −22 mA to −24 mA before slowly decreasing to −19 mA. Filling the “branched” pores of our alumina template with metallic Cu requires about 21 C of charge and about 15 min in the working conditions detailed in the Materials and Methods section. Attempts to electrochemically deposit P3HT directly onto Cu were characterized by lack of reproducibility, most probably because of the oxidation of Cu at the high potentials required for the electrooxidation of the 3HT monomer. Therefore, a short segment of Au was electrochemically deposited onto the Cu before the deposition of the P3HT segment.

As shown in Figure 2B, the electrochemical deposition of the metallic Au into the cylindrical pores of our alumina template initially produced a current of about 0.4 mA. Then, the current increased slightly and stabilized around 0.5 mA. Obtaining Au segments of appropriate length (i.e., segments which protect well the underlying Cu layers during the oxidative polymerization of 3HT) requires about 2 C of charge and about 60 min in the working condition detailed in the Materials and Methods section. Preliminary experiments have shown that electrochemically synthesized P3HT films give larger photocurrents when they are synthesized in the presence of TBAHFP than when they are synthesized in the presence of LiClO_4_. In other words, doping P3HTs films with HFP^-^ anions instead of ClO_4_^−^ anions increases the photosensitivity of P3HT films (data not shown). Therefore, we decided to synthesize P3HT nanowires in the presence of TBAHFP. Figure 2C shows the two voltammograms recorded during the electrochemical synthesis of the P3HT nanowires. The second voltammogram is characterized by larger currents than the first one, indicating that the P3HT formed during the first potential cycle is characterized by good electrical conductivity and, thus, increases the electrochemically active area of the working electrode. The second voltammogram also shows two overlapping current peaks due to the oxidation of the just formed P3HT and two overlapping current peaks due to the reduction of the just formed P3HT. Somewhat better-defined current peaks are found at 0.85 V (for the oxidation of P3HT) and at 0.32 V (for the reduction of P3HT). Current peaks due to the oxidation and the reduction of P3HT were previously observed to appear at lower potentials when the molecular weight of P3HT increases [31]. Therefore, the overlapping current peaks observed in the voltammograms recorded during the synthesis of the P3HT nanowires (Figure 2C) might be due to the coexistence of polymers with different molecular weights. After the electrochemical polymerization of 3HT to P3HT, the sacrificial metal layers were etched away, and the porous alumina template dissolved (as detailed in the Materials and Methods section). Centrifugation and washing steps were then used to obtain 1 mL of P3HT nanowire suspension in ethanol or water. The typical orange color of this suspension was the first indication that P3HT nanowires were successfully synthesized.

The uniqueness of the template-assisted, electrochemical synthesis of P3HT nanowires relies on the fact that polymerization of P3HT, doping of P3HT, and nanostructuring of P3HT advantageously occurs in the very same time (in contrast with other nanostructuring methods which rely on previously synthesized P3HT as mentioned also in the Introduction). In addition to its relative simplicity, the template-assisted, electrochemical synthesis of P3HT nanowires also provides good control over the length of the obtained P3HT nanowires. The length can be controlled in two ways. First, it can be controlled via the concentration of the 3HT monomer used in the electrochemical synthesis of P3HT. Second, it can also be controlled by the electrolysis time (e.g., by the number of potential cycles when using cyclic voltammetry for the synthesis). To demonstrate this advantage of the template-assisted, electrochemical synthesis of P3HT nanowires, we have synthesized “long” P3HT nanowires by using a 3HT concentration of 30 mM and also “short” P3HT nanowires by using a 3HT concentration of 11 mM (see the next paragraph).

### 3.2. Characterization of the P3HT Nanowires

**Figure 3:**
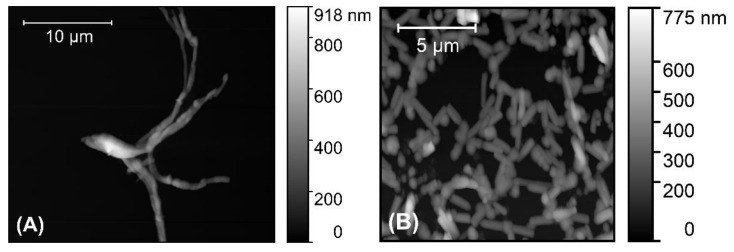
AFM images of “long” P3HT nanowires (**A**) and of “short” P3HT nanowires (**B**).

AFM was used to investigate P3HT nanowires drop-casted onto glass coverslips. The obtained AFM images (such as those shown in Figure 3) revealed that the P3HT nanowires synthesized using a solution of 30 mM 3HT are clearly much longer than those obtained using a solution of 11 mM 3HT. Thus, the concentration of the 3HT monomer used during the electrochemical synthesis of the P3HT nanowires allows for controlling the length of these nanowires in an easy, convenient way.

**Figure 4:**
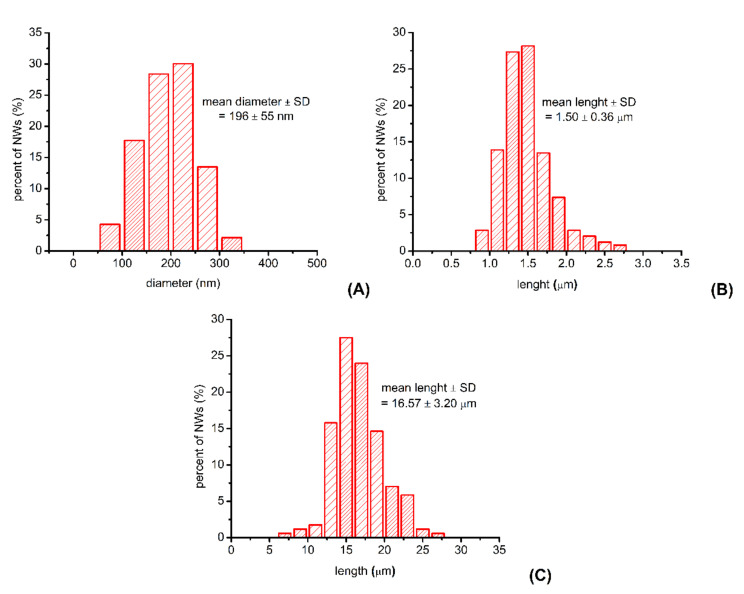
Distribution of P3HT nanowire diameters and lengths. The distribution of the nanowire diameters (**A**) was obtained by analyzing the AFM images of 140 nanowires from eight different batches. The distribution of the lengths of “short” P3HT nanowires (**B**) was obtained by analyzing the AFM images of 240 nanowires from five different batches. The distribution of the lengths of “long” P3HT nanowires (**C**) was obtained by analyzing either AFM or optical microscopy images of 170 nanowires from three different batches.

AFM images were also used to evaluate the diameter and the length of the synthesized P3HT nanowires. The average diameter of the synthesized P3HT nanowires was found to be 196 ± 55 nm, a value that is a good match with the diameter of the cylindrical pores of the alumina template used in our experiments (i.e., 200 nm according to the producer). The distribution of the diameters of the synthesized P3HT nanowires is shown in Figure 4A. As one can observe, about two-thirds of all nanowires have diameters ranging between 150 and 250 nm. Only about 10% of all nanowires have diameters either smaller than 100 nm or larger than 300 nm. This relatively wide distribution of the nanowire diameters is likely due to the nonuniformity of the cylindrical pores of the alumina template [32], and is also due to the way the diameters were measured (e.g., the diameter of a nanowire that does not lay perfectly flat on the glass substrate will be overestimated when measured from an AFM image). The average length of the “short” P3HT nanowires was found to be 1.50 ± 0.4 µm, while the average length of “long” P3HT nanowires was found to be 17 ± 3 µm. The distributions of the lengths of the different P3HT nanowires are shown in Figure 4B,C.

**Figure 5:**
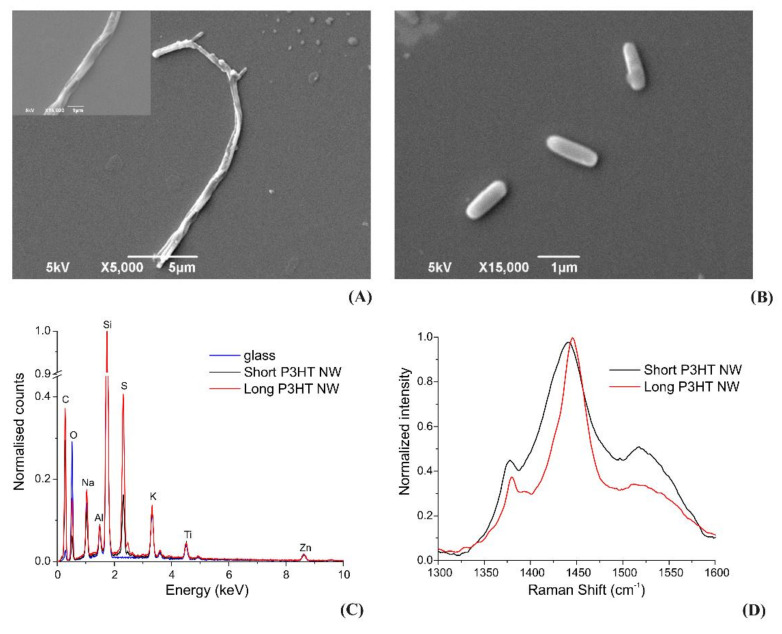
SEM, EDS and Raman spectroscopy characterization of the nanowires. (**A**,**B**) show representative SEM images of “long” and “short” nanowires, respectively. (**C**) EDS spectra showing the evident K_α_ peak of sulfur (S) from P3HT and no other elements apart from the contribution of the glass coverslip substrate, whose spectrum is also presented. (**D**) Raman spectra acquired at 633 nm (1 s) showing peaks at ~1380 and 1440 cm^−1^, due to C–C intra-ring stretching and C=C symmetric stretching, respectively, both of them characteristic for P3HT.

We characterized the nanowires drop-casted on glass coverslips also by SEM. The obtained SEM images (see Figure 5A,B) show nanowire shapes and dimensions in agreement with those observed in the AFM images. The SEM images of the “long” P3HT nanowires reveal, just as the AFM images, the increased tendency of these nanowires to form aggregates in which several wires are intertwined.

An EDS investigation was also carried out to evaluate the presence of elements which might have contaminated the nanowires during their synthesis and processing. Figure 5C compares the X-ray spectra from “short” and “long” nanowires, both presenting a strong sulfur K_α_ peak from P3HT. A background of various other elements reflects the composition of the glass coverslip on which the nanomaterials were deposited.

Raman spectra were also acquired for both “short” and “long” nanowires by mapping the samples over XY in order to identify the regions with high concentrations of nanomaterials to improve the signal to noise ratios (Figure 5D). The reported spectra represent the regions with the highest nanowires concentrations. The spectra of both experimental groups feature the main characteristic P3HT peaks at 1380 and 1440 cm^−1^ (due to C–C intra-ring stretching and C=C symmetric stretching, respectively), confirming the quality of the analysed material.

**Figure 6:**
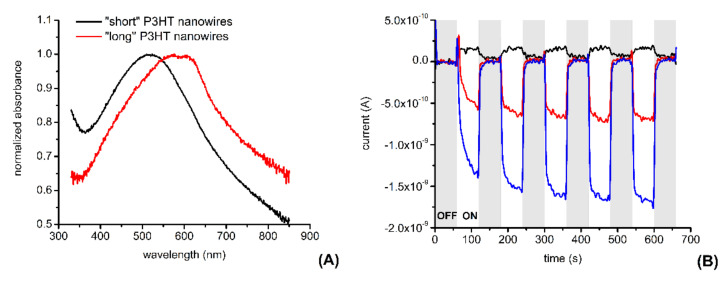
Typical absorbance spectra of suspensions containing P3HT nanowires (**A**) and photocurrents observed with a P3HT nanowires-modified glassy carbon electrode (**B**). Normalized absorbance spectra are shown for suspensions with either “short” or “long” P3HT nanowires. Photocurrents are shown for the bare glassy carbon electrode (black signal), for the glassy carbon electrode modified by drop-casting with 18 µL of “short” P3HT nanowire suspension (red signal), and for the glassy carbon electrode modified by drop-casting with 36 µL of “short” P3HT nanowire suspension (blue signal). See the Materials and Methods section for additional experimental details.

The UV-vis spectra of the suspensions containing electrochemically synthesized P3HT nanowires show interesting differences in between “long” and “short” P3HT nanowires (see Figure 6A).

The spectrum of the suspension containing “short” P3HT nanowires shows an absorption peak centered around 516 nm (i.e., shows an absorption peak that is red shifted in comparison with the absorption peak of a homogenous P3HT solution, which is usually centered around 455 nm [33]). Meanwhile, the spectrum of the suspension containing “long” P3HT nanowires (resembles the spectrum of P3HT films and) shows two additional absorption peaks at 570 nm and at 609 nm. The observed red shift is usually attributed to an increase in π−π stacking of the polymer chains leading to an increase in interchain coupling [13,14].

We also investigated whether P3HT nanowire-modified electrodes produce photocurrents when illuminated or in dark conditions. Figure 6B shows that, compared to unmodified glassy carbon electrodes, glassy carbon electrodes modified with “short” P3HT nanowires produce significantly larger currents following illumination with white light at a power density of ~1.3 mW cm^−2^. Moreover, the photocurrents produced by the P3HT nanowire-modified glassy carbon electrode are proportional with the amount of nanowire deposited onto the surface of the electrode (indicating the P3HT nanowires as the source of these photocurrents).

The above results establish template-assisted, electrochemical synthesis as a relatively easy procedure to produce photosensitive P3HT nanowires of different and controlled lengths. Next, we moved to the investigation of the compatibility of such nanowires with neurons. While the good biocompatibility of P3HT itself was already demonstrated (e.g., in [34]), the TBAHFP that is entrapped into our P3HT nanowires during their synthesis, and the species which might adsorb onto the nanowires (e.g., during the etching of the sacrificial metal layers and during the dissolution of the alumina template) might still alter the basic biocompatibility of P3HT. A good biocompatibility of the P3HT nanowires is a very important requirement of their use in neural interfaces.

### 3.3. Interfacing the P3HT Nanowires with Neurons

**Figure 7:**
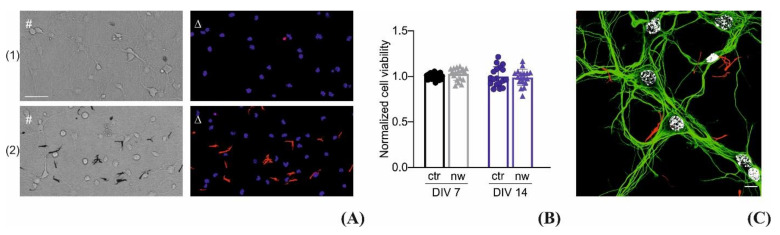
Neuronal viability. Representative bright field (#) and epifluorescence images (∆) of neurons grown in the presence of P3HT nanowires (2 or under control conditions (1). P3HT nanowires are black and red in the transmitted and epifluorescence images, respectively, thanks to their optical properties. Cultures were stained with Hoechst-33342 for nuclear visualization (blue) and propidium iodide (PI, red) for cell death quantification (scale bar: 100 µm) (**A**). Cell viability was evaluated by fluorescence microscopy at DIV7 and DIV14. The number of PI-positive cells was subtracted to the total number of Hoechst-positive cells to obtain the number of living cells. The percentages of live neurons with respect to the total number of Hoechst-positive cells was assessed for each experimental group. Viability values were normalized to the respective average value of the control sample and plotted as Means ± SEM with superimposed individual experimental points. No significant changes in cell viability were observed under the various experimental conditions (unpaired Student’s *t*-test, *n* = 18 fields per experimental condition, from two independent neuronal preparations) (**B**). Representative confocal microscopy image showing primary mouse cortical neurons (grown onto poly(l-lysine)-coated glass further modified with endogenously fluorescent P3HT nanowires (red)) immunolabeled for β3 tubulin (green) and with Hoechst-33342 (white) (scale bar: 10 µm) (**C**).

“Long” P3HT nanowires were incubated with primary neuronal cultures to assess their biocompatibility. Before incubation, the nanowires suspensions had been dialyzed (using a dialysis membrane with a MWCO of 3.5 kDa) against 0.8 L absolute ethanol to remove any residues from the template-assisted electrochemical synthesis procedure. Glass coverslips were first treated with adhesion molecules and then with P3HT nanowires as described in the Materials and Methods section. Once the ethanol of the P3HT nanowire suspension evaporated, the standard neuron plating procedure was adopted with no additional washing or treatment. Cultures were incubated for either 7 or 14 days in vitro (DIV) and tested for viability as shown in Figure 7A, in parallel with preparation treated with vehicle. The representative brightfield images depict the P3HT nanowires as black, thanks to their high absorbance in the visible range, while when excited with green light their intrinsic photoluminescence is in the red region of the spectrum. The cell viability quantified in Figure 7B showed no significant differences among the experimental groups at both DIV, proving the suitability of the nanomaterials as interfaces for primary neuronal cultures. Moreover, Figure 7C depicts 14 DIV neurons, treated with nanowires (red) and immunolabeled for β3 tubulin (green) and nuclei (white), showing once more that neurons were able to grow and develop a network without any detectable interference from the presence of the P3HT nanowires.

**Figure 8:**
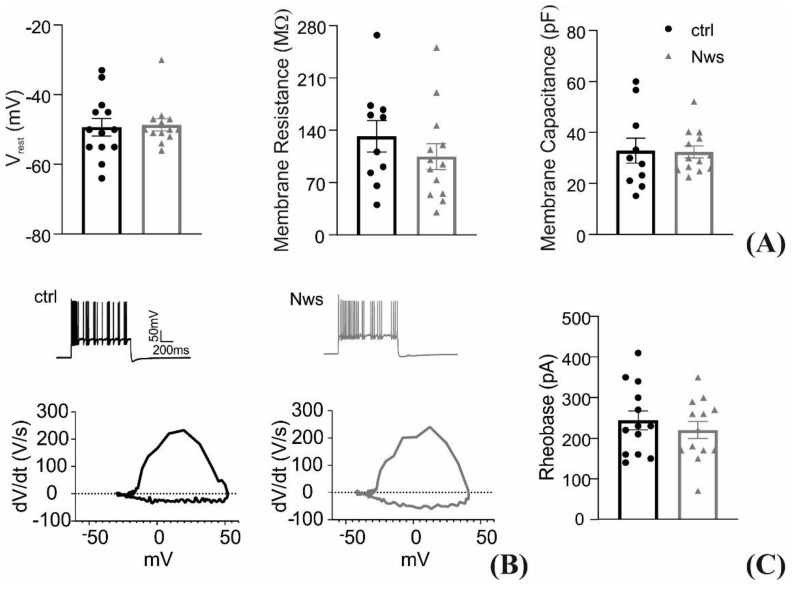

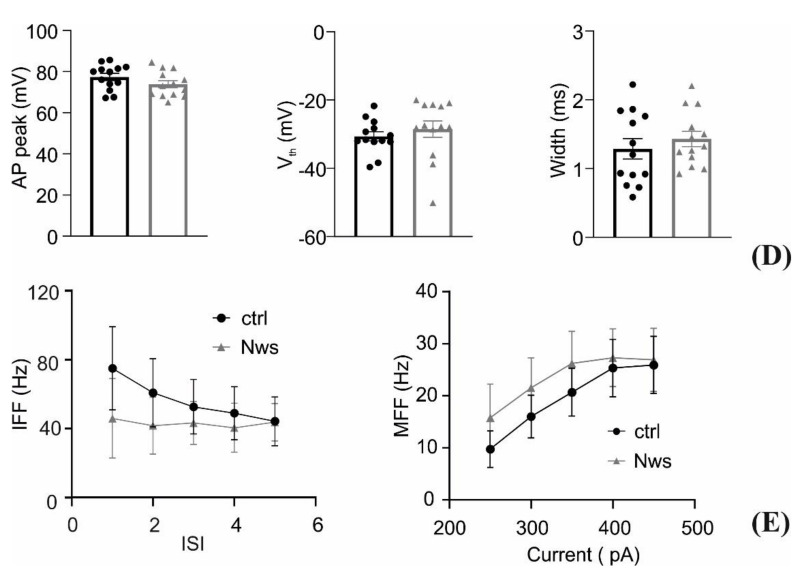
Electrophysiological properties of primary neurons grown in the presence of P3HT nanowires. From left to right: resting membrane potential, membrane resistance, membrane capacitance (**A**). Representative traces of AP firing evoked by 350 pA current injection under both experimental conditions, and representative phase-plane plot analysis (dV/dt vs. V) of AP waveform obtained under control conditions (left) and in the presence of P3HT nanowires (right) (**B**). Minimal current injection intensity necessary to evoke an AP (rheobase) (**C**). From left to right: peak, threshold potential (V_th_), and width of AP (**D**). Instantaneous firing frequency (IFF) at each inter-spike interval (ISI) evoked by the injection of 350 pA depolarizing current (left) and mean firing frequency (MFF) plotted as a function of the injected current (right) (**E**). All data are shown as Means ± SEM with superimposed individual experimental points. All the analyzed parameters were not significantly different between the two experimental groups; Student’s *t*-test (**A**–**D**) and two-way ANOVA with Bonferroni’s multiple comparison tests (**E**); *n* = 9–13 cells, from two independent neuronal preparations).

The same neuronal cultures were also subjected to an electrophysiological analysis by patch-clamp experiments. Figure 8A depicts that the passive membrane properties of the neurons incubated with P3HT nanowires do not differ from the ones of control preparations. Neuronal excitability was also assessed as show in Figure 8B, where the representative current-clamp firing patterns and the action potential (AP) phase-plane plot demonstrate no interference of the P3HT nanowires with the biological mechanisms of intrinsic excitability. The quantification of resting membrane potential, the threshold voltage, and AP duration further demonstrate no significant differences between the two experimental groups (Figure 8C). Finally, the characterization of the firing rate and its dynamics confirms once more that the P3HT nanowires are a fully biocompatible tools for neuronal interfacing (Figure 8D), paving the way for their potential application as photostimulation tools.

In order to provide a better context for the present work, it is important to mention that, in addition to P3HT, a number of other photosensitive materials were also used to develop interfaces for the photostimulation of neurons. For example, blends of semiconductor nanocrystals (such as CdSe/CdS core/shell nanorods) and carbon nanotubes were used to provide light sensitivity to light-insensitive chick retinas [35]. Gold-nanoparticle decorated titania was also successfully used to restore the photosensitivity of retinal neurons both in vitro and in vivo [36]. Gold nanorods were coupled with an antibody against temperature-sensitive ion channels and used to induce photosensitivity to light-insensitive retinal cones expressing temperature-sensitive ion channels [37]. Investigating how our P3HT nanowires compare to these nanomaterials is beyond the aims of the present study.

## 4. Conclusions

Template-assisted, electrochemical synthesis was found to be a promising method of synthesizing P3HT nanowires. The fact that it combines polymerization, doping, and polymer nanostructuring into one, relatively simple step represents the most important advantage of the method. The possibility of easily tuning the length of the produced nanowires represents another important advantage of the method. P3HT nanowires produced photocurrents following illumination. The photocurrents were found to be proportional with the areal number density of the P3HT nanowires on the working electrode. To exploit such nanomaterials for photostimulation of neuronal cells, we proved their biocompability upon incubation for different periods with primary neuronal cultures. We demonstrated that their presence does not affect the membrane properties of the neurons or the excitability of the neurons as evaluated by patch-clamp experiments. These results show the potential of the described synthesis methodology to fabricate injectable P3HT-based photosensitive nanowires with high biocompatibility, ultimately paving the way for their exploitation for neuronal photostimulation.

## Data Availability

Data generated during the present study are available from the corresponding authors on reasonable request.
